# Supplementary material for: A photoelectrochemical platform for the capture and release of rare single cells
Source: Nat Commun. 2018 Jun 12;9:2288. doi: 10.1038/s41467-018-04701-y (PMC5997639; doi:10.1038/s41467-018-04701-y)
Supplement: Supplementary file 1 — Supplementary Information [file 41467_2018_4701_MOESM1_ESM.pdf]

Supplementary Information

A photoelectrochemical platform for the capture and release of rare single cells

Stephen G. Parker, Ying Yang, Simone Ciampi, Bakul Gupta, Kathleen Kimpton, Friederike M. Mansfeld, Maria Kavallaris, Katharina Gaus, J. Justin Gooding

Surface fabrication

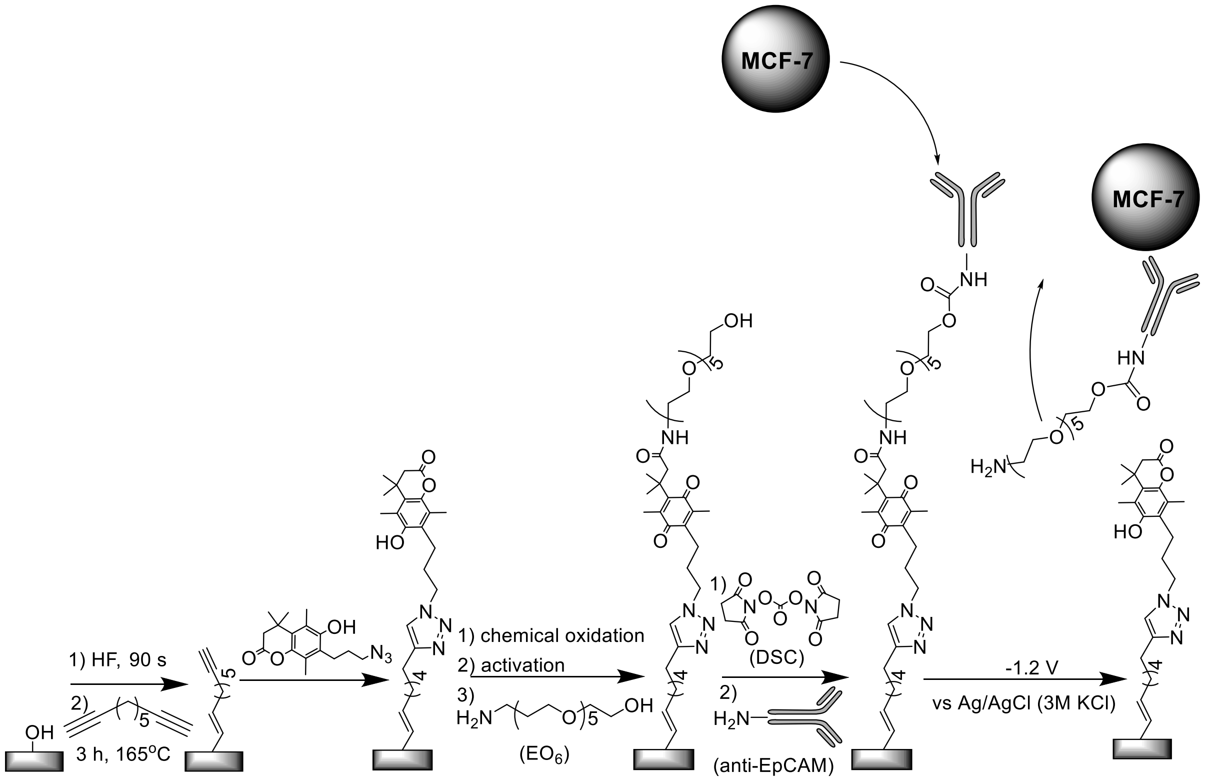

Supplementary Figure 1. Surface modification to capture & release. A schematic showing how a biological receptor molecule (anti-EpCAM) was tethered to a silicon surface via a switchable component. The receptor molecule then binds to EpCAM antigens overexpressed in the MCF-7 cells where a negative bias vs Ag|AgCl can be applied to the surface to initiate switching of the switchable component to recover the MCF-7.

Acetylene-functionalised silicon(100) surface

Assembly of the acetylenylated Si(100) was achieved by covalently attaching 1,8-nonadiyne to the Si(100) surface in a process known as hydrosilylation, that has been reported previously in the literature[5,6] and optimised by Gooding and co-workers[7-10]. 1,8-Nonadiyne **1** was added to a flame-dried Schlenk flask (a custom-made flask designed specifically for this procedure). The 1,8-nonadiyne was degassed by freeze-pump-thaw cycles until no more bubbles were observed in the 1,8-nonadiyne upon thawing and then stored under argon. A Si(100) wafer was cut into the appropriate size using a diamond cutter, cleaned thoroughly with DCM, then with piranha solution (2:1 v/v mixture of sulfuric acid and hydrogen peroxide. **Caution:** reacts violently with organic materials) at 90 °C for 40 min and then rinsed with copious amounts of MilliQ™ water. The wafer was then etched with 2.5% hydrofluoric acid for 90 s, dried with filter paper and then under an argon stream before being placed into the Schlenk flask containing the 1,8-nonadiyne. A gentle argon stream was added to the Schlenk flask whilst it was immersed for 3 h in an oil bath set to 165 °C. After the reaction was complete, the sample was cleaned thoroughly with DCM and then blown dry under a stream of argon before being characterised or further modified with the redox-sensitive linker molecule.

Attachment of the redox-sensitive linker 12 to the acetylenyl surface

In a typical click procedure, to a reaction vial containing the alkyne-functionalised silicon surface were added (i) the azide **12** (Supplementary Figure 3) or 3-azidopropionic acid **14** (Supplementary Figure 10) (10 mM, degassed 2-propanol/water, 2:1), (ii) copper(II) sulfate pentahydrate (1 mol% relative to the azide) and (iii) sodium ascorbate (60 mol% relative to the azide). Reactions were carried out at 35 °C, in the dark without excluding air from the reaction environment and stopped after 3 h by removal of the modified sample from the reaction vessel[11]. The prepared surface-bound [1,2,3]-triazoles samples were rinsed consecutively with copious amounts of ethanol and water, left in 0.5 M hydrochloric acid for

1 min to remove excess copper and then consecutively washed again with copious amounts of water, ethanol and dichloromethane before being either analyzed or further reacted.

Oxidation of confined lactone moieties to benzoquinone acids

Lactone-functionalised surface samples were rinsed with tetrahydrofuran (ca. 50 mL) and then transferred to a mixture of tetrahydrofuran (6 mL) and water (3 mL). *N*-Bromosuccinimide (160 mg, 90 mmol) was added to the reaction vial and the solution agitated for 3 h in the dark at room temperature. The putative ring-opened, benzoquinone acid intermediate was then copiously rinsed with tetrahydrofuran and water before being analyzed or EDC/NHS-activated.

Activation of the terminal acid group with coupling chemistry

EDC (200 mg, 1.04 mmol) and NHS (240 mg, 2.09 mmol) were dissolved in water (10 mL). The acid-terminated silicon surface was added to the solution and the uncapped flask was left in the dark at room temperature with frequent shaking to remove air bubbles that were formed on the surface. The activated[12] silicon samples were removed from the reaction mixture when no bubbles were observed on the surface after 20 mins and were washed with copious amounts of water before being further reacted.

General procedure for the reactions of activated samples with nucleophiles

Activated benzoquinone samples were washed with DMF (ca. 20 mL), blown dry under a stream of argon and placed into a reaction tube containing (OEO6) (0.1 M) in DMF (4 mL) and DMAP (2.0 mg, 0.02 mmol). The reaction was continued in the dark at room temperature for 16 h and stopped by removing the silicon wafer from the reaction mixture. Samples were washed with DMF, water and DMF consecutively and were analyzed or further reacted.

Disuccinimidyl carbonate coupling of the hydroxyl-terminated surface.

OE moiety-terminated silicon samples were placed into a reaction tube containing: a) DSC (0.1 M) and b) DMAP (0.1 M) in dry acetonitrile in the dark at room temperature for 20 h. The active carbonate samples were then rinsed with ethyl acetate, DCM and ethanol and dried under a stream of argon for further modification.

Antibody-conjugation on DSC-activated OEO-terminated surfaces

A 1:200 (v/v) antibody solution in 1X DPBS was dropped onto parafilm (50 μL/cm^2 silicon) and the DSC-terminated silicon surface was placed, upside down, on top of the antibody mixture. The surface was left to incubate in the dark at room temperature for 1.5 h before being rinsed with MilliQ™ water and 1X DPBS with 0.5% (v/v) Tween 20 and stored in 1X DPBS for analysis or cell capture.

Surface characterisation

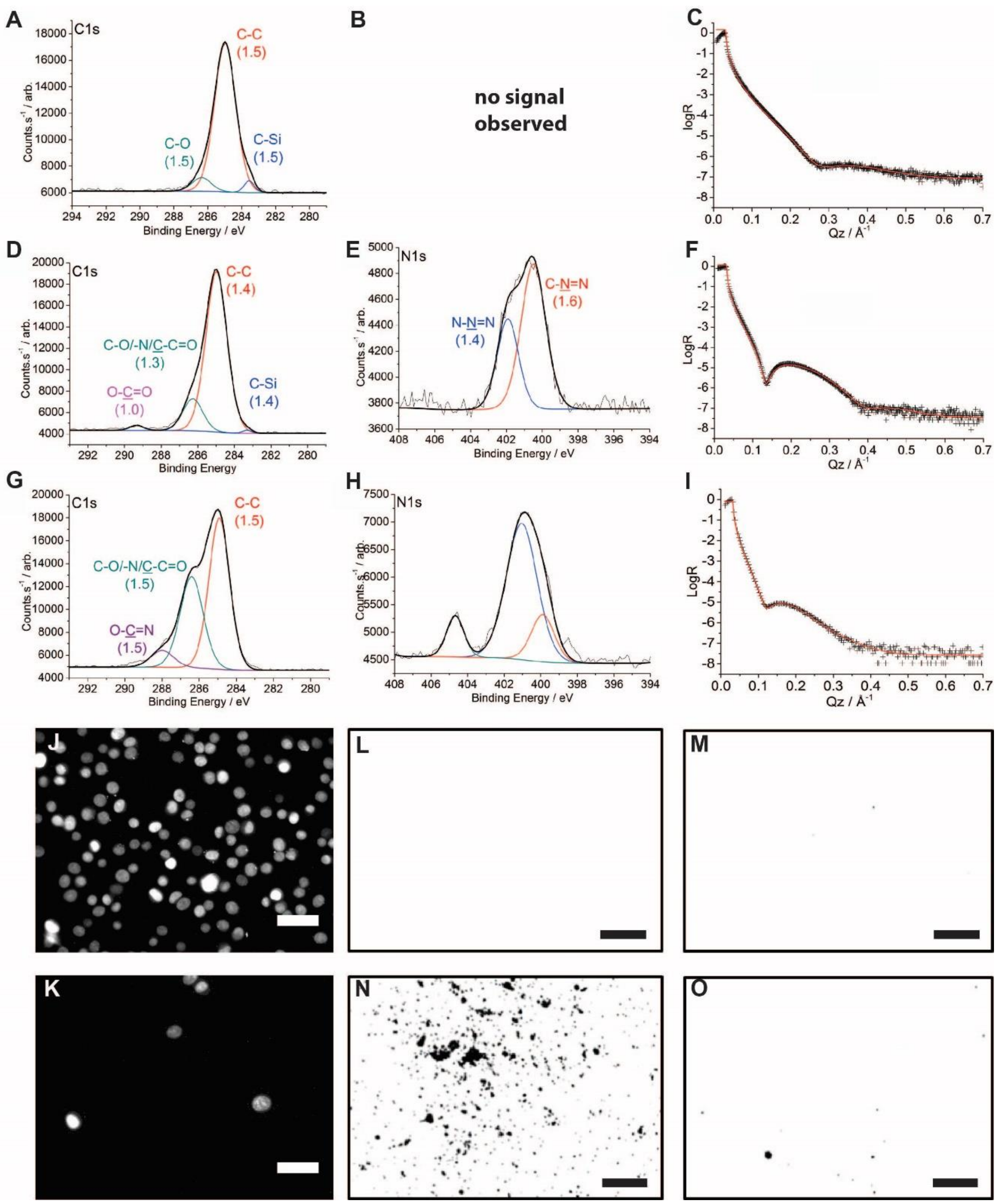

Supplementary Figure 2. Surface characterisation of capture & release surface. C1s narrow scans for A) nonadiyne-modified surface, D) switchable linker molecule-modified surface and G) N_3-OEO4-NH_2-terminated silicon surface. N1s narrow scans for B) nonadiyne-modified surface, E) switchable linker molecule-modified surface and H) N_3-OEO4-NH_2-terminated silicon surface. Reflectometry spectra for C) nonadiyne-modified surface, F) switchable linker molecule-modified surface and I) N_3-OEO4-NH_2-terminated silicon surface. Fluorescence micrographs of MCF-7 cells (15 000 cells mL^{-1}, 1 mL) plated on a 1 cm^2 silicon surface modified with J) an OEO4 and K) an OEO6. Upon capture, cells were stained with Hoechst 33342. Images were recorded

with a 40X objective, 500 ms exposure and a gain factor of 2 dB. Scale is the same for both images (50 µm). Fluorescence micrograph of L) an OEO6-terminated Si(100) surface, M) an OEO6-terminated surface incubated in Cy3™-conjugated donkey anti-mouse IgG antibodies without the DSC activator, N) antibody-conjugated surface and O) a switched surface. Images were recorded with a 40X objective, 2000 ms exposure and a gain factor of 2 dB. Images were converted to 8-bit from RGB and then thresholded. Scale is the same for all images (200 µm)

For the passivation of the surface with the 1,8-nonadiyne layer, X-ray photoelectron spectroscopy (XPS) reveals a dominant C-C bond at 285 eV, as well as small shoulders at 283 and 286 eV, which correspond to the C-Si bond and hydrolysis of the C=C bond at the proximal end of the layer, respectively[7]. This provides evidence for the presence of the passivating layer on the surface. Importantly, the absence of other carbon species alludes to the absence of any contaminants present on the surface. Modelling of X-ray refelectrometry (XRR) (Supplementary Figure 2C) data revealed that the monolayer had a thickness of 10 Å, consistent with a 1,8-nonadiyne layer sitting on the surface at a 60° angle. The passivating layer is subsequently modified by an electrochemically cleavable linker *via* a 1,3-dipolar Huisgen click reaction. The C1s narrow scan (Supplementary Figure 2D) reveals a 1:6 atomic composition for the peaks at 290 eV and 286 eV, respectively. The peak at 286 eV is attributed to carbons bonded to a single oxygen atom or nitrogen atom, as well as being adjacent to a carboxylic acid group whilst the peak at 290 eV is attributed to a carbon bonded to two oxygen atoms[7]. This ratio aligns with the expected ratio within the electrochemically-cleavable molecule and provides evidence for its presence on the surface. The N1s narrow scan (Supplementary Figure 2E) illustrates two peaks at 400 and 402.5 eV with an atomic ratio of 2:1 respectively. These peaks are indicative of a triazole group on the surface[13]. Importantly, the absence of a peak at 405 eV, characteristic of the central nitrogen atom within an azide group[14], highlights the absence of non-specifically adsorbed electrochemically-cleavable moieties. By comparing the observed carbon to nitrogen atomic

composition with the theoretical carbon to nitrogen ratio for varying coupling efficiencies, a coupling efficiency of 56% was calculated for this step. XRR (Supplementary Figure 2F) reveals an increase in thickness from 10 to 22 Å after this step, expected with the theoretical thickness for this surface. Oxidising the cleavable molecule results in the opening of the ring and reveal a terminal carboxylic acid terminal moiety. An OEO containing four repeating units, an amine at the proximal end to attach to the succinimidyl ester on the surface and an azide group at the distal either end was used to monitor the coupling of this molecule to the activated cleavable linker molecule. The presence of the distal azide group serves as a useful group to be monitored with XPS. A C1s narrow scan (Supplementary Figure 2G) reveals a significant increase in a peak at 287 eV, which correlates to the C-O bonds within the OEO. Furthermore, a N1s narrow scan (Supplementary Figure 2H) reveals a peak at 405 eV, which correlates to the central nitrogen atom within the distal azide group. XPS reveals the presence of the OEO group on the surface. Despite the azide group being highly observable in XPS, it would be more suitable for bioconjugation if the OEO was terminated with a carboxyl or hydroxyl group. These groups can be activated with succinimides that can be nucleophilically substituted with amines, that are ubiquitous within biological receptor molecules. For this reason, an OEO containing a hydroxyl group in place of an azide group was tethered to the cleavable linker molecule. Modelling of XRR on an OEO-terminated surface (Supplementary Figure 2I) revealed an increase in the surface thickness from 22 to 25 Å and provides further evidence for the conjugation of the OEO to the cleavable-terminated surface. It is worth noting that XPS scans were similar to those observed for the cleavable linker-modified surface after applying a negative bias to the surface. The next step was to optimise the length of the antifouling molecule to obtain suitable antifouling properties of the surface. Supplementary Figure 2J illustrates that high levels of non-specific adsorption of MCF-7 cells was observed when the surface was modified with an OEO of four repeating units.

However, a significant decrease in the amount of non-specific adsorption of MCF-7 cells was observed when an OEO of six repeating units was used, highlighting the importance to optimise the length of the antifouling layer that is used (Supplementary Figure 2K). For this reason, 17-amino-3,6,9,12,15-pentaoxaheptadecan-1-ol (OEO6) was attached to the cleavable species to prevent non-specific adsorption of contaminating cells. Finally, the antibodies to pre-concentrate these target cells were bioconjugated to the OEO6 species *via* a *N,N*-disuccinimidyl carbonate activating group. To monitor the conjugation of the antibodies to the OEO6 layer, a Cy3-tagged antibody was tethered to the surface. A fluorescence micrograph (Supplementary Figure 2L) revealed no background fluorescence of the OEO-terminated silicon surface. Similarly, low levels of fluorescence was observed when the Cy3-labelled antibodies were incubated on the unactivated OEO-terminated surface (Supplementary Figure 2M), showing the ability of the surface to prevent the adsorption of non-specific antibodies. However, a significant increase was observed when the antibodies were covalently attached to the activated OEO-terminated silicon surface (Supplementary Figure 4N). Interestingly, lower levels of fluorescence was observed after a negative bias (-2.3 V *vs* Ag|AgCl (3 M KCl)) was applied to the surface from Supplementary Figure 2N, alluding to the capability of the surface to release the captured cells (Supplementary Figure 2O).

Synthesis of electrochemically-cleavable linking molecule

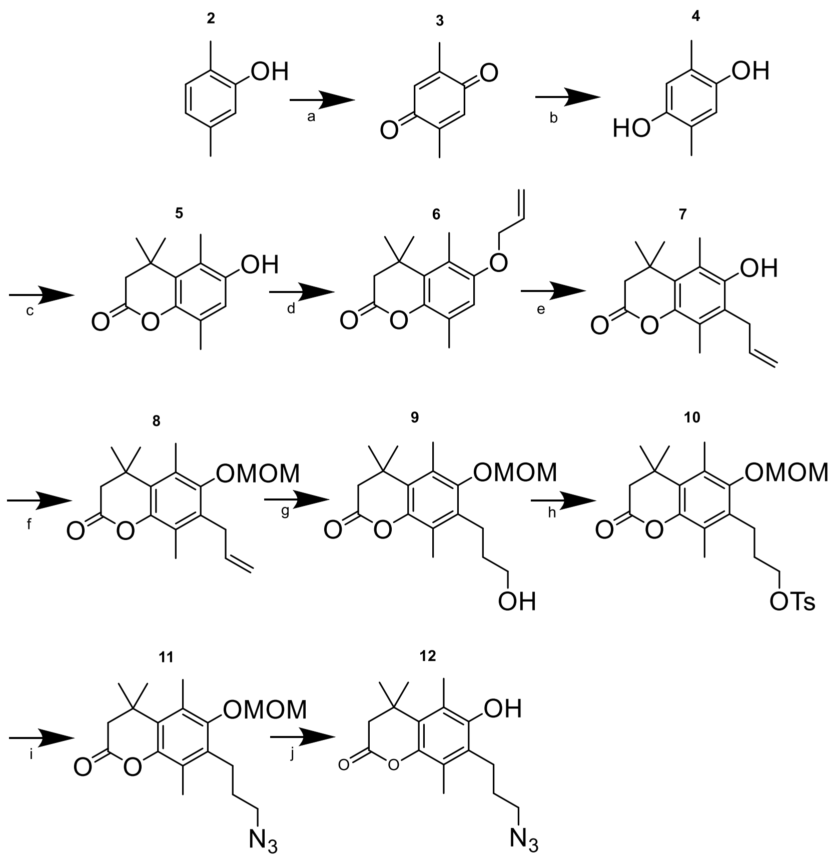

Supplementary Figure 3. Synthesis of the redox-sensitive lactone linker 12. a. Na_2CrO_4, H_2SO_4/Et_2O, 36%; b. $Na_2S_2O_4$, 94%; c. β,β-dimethylacrylic acid, CH_3SO_3H, 81%; d. allyl bromide, K_2CO_3, NaI, 83%; e. BCl_3-heptane, 79%; f. MOMCl, DIPEA, 92%; g. BH_3-THF, NaOH/H_2O_2, 44%; h. TsCl, Py, 40%; i. NaN_3, 76%; j. CBr_4, 70%.

*(a) 2,5-Dimethylbenzoquinone (**3**)*

The alkylquinone **3** was prepared from 2,5-dimethylphenol **2** in a one-pot procedure *via* a two-phase Jones oxidation reaction according to literature procedures[1] with minor modifications. 2,5-Dimethlyphenol **2** (30.0 g, 246 mmol) (Sigma-Aldrich, 99%) was dissolved in diethyl ether (*ca*. 400 mL) and the solution cooled to 0 °C in an ice/water bath. The reaction flask was equipped with a large magnetic stirrer and a dropping funnel. The Jones reagent was prepared in a separate flask from sodium dichromate dihydrate (150 g, 503 mmol) (Alfa Aesar, ACS grade) by adding it to room temperature water (155 mL) before cooling the solution in an ice/water bath. At this time, ice-cold 96% sulfuric acid (68 mL)

(Ajax Finechem, analytical grade) was added with vigorous shaking and the mixture was transferred to the dropping funnel. Addition of the Jones reagent to the phenol **2** solution was done dropwise over a 2 h period while stirring. After the addition, the ice bath was removed and stirring was continued at room temperature for a 36 h period. The reaction mixture was transferred to a separation funnel and extracted with diethyl ether (4 × 100 mL). The combined organic extracts were washed with water (200 mL), sodium bicarbonate solution (2 × 100 mL) and brine (100 mL) and dried over magnesium sulfate ($MgSO_4$) (Ajax Finechem, analytical grade). Filtration through celite (Sigma-Aldrich) and evaporation of the solvent *in vacuo* afforded crude quinone **3** as a dark orange solid. Column chromatography (toluene) gave the quinone **2** as a yellow powder (12.1 g, 36%). ^1H NMR (300 MHz, $CDCl_3$) δ: 6.60 (q, 2H), 2.05 (s, 6H); ^{13}C NMR (75.5 MHz, $CDCl_3$) δ: 188.16, 145.92, 133.49, 15.61; IR (KBr, cm^{-1}): 1665, 1643, 1611, 1438, 1380, 1349, 1254, 1154, 1005, 927, 796.

*(b) 2,5-Dimethylhydroquinone (**4**)*

Reduction of the alkyl quinone **3** to the corresponding hydroquinone **4** was done according to the literature procedure[2]. 2,5-Dimethylbenzoquinone **3** (11.8 g, 86.4 mmol) was dissolved in diethyl ether (150 mL) and the solution obtained was transferred to a separation funnel previously loaded with sodium hydrosulfite (110 g, 632 mmol) (Sigma-Aldrich, ACS grade) and water (110 mL). The mixture was shaken until the organic layer had turned from a vivid yellow to a pale yellow colour. The organic layer was then collected, and the aqueous phase extracted with diethyl ether (3 × 40 mL). The pooled organic phase was washed with brine (2 × 40 mL), dried over $MgSO_4$, filtered and dried *in vacuo* to give the quinone **4** (11.2 g, 94%) as a white powder used in the successive step without further purification. ^1H NMR (300 MHz, DMSO-d_6) δ: 8.32 (s, 2H), 6.44 (s, 6H), 1.99 (s, 6H); ^{13}C NMR (75.5 MHz, DMSO-d_6) δ: 147.46, 121.17, 116.87, 15.77; IR (KBr, cm^{-1}): 3246, 1427, 1189, 1186, 868, 827, 688.

*(c) 6-Hydroxy-3,3,5,8-tetramethylhydrocoumarin (**5**)*

The alkylhydroquinone **4** was converted to the lactone **5** by reaction with *β,β*-dimethylacrylic acid in a Friedel-Craft type addition reaction[2,3]. *β,β*-Dimethylacrylic acid (8.7 g, 87.2 mmol) (Sigma-Aldrich, 97%) was added to a solution of 2,5-dimethylhydroquinone **4** (10.9 g, 79.0 mmol) in methanesulfonic acid (*ca.* 125 mL) (Alfa-Aesar, 98+%) and the obtained solution stirred for 3 h at 70-80 °C under an argon atmosphere. The crude reaction mixture was allowed to cool to room temperature and subsequently ice (*ca.* 150 g) was slowly added into the reaction flask to give a grey suspension which was allowed to warm to room temperature before being extracted with ethyl acetate (3 × 40 mL). The pooled organic layers were washed with water (2 × 40 mL), saturated sodium bicarbonate solution (2 × 40 mL) and then dried over $MgSO_4$. Upon filtration and evaporation *in vacuo* a white residue was obtained. The crude title compound was dissolved in a minimum amount of warm ethyl acetate and crystallisation upon the addition of ice-cold hexane yielded the coumarin **5** as an off-white powder (14.1 g, 81%). ^{1}H NMR (300 MHz, $CDCl_3$) δ: 6.57 (s, 1H), 5.00 (s, 1H), 2.57 (s, 2H), 2.33 (s, 3H), 2.22 (s, 3H), 1.46 (s, 6H); ^{13}C NMR (75.5 MHz, $CDCl_3$) δ: 168.63, 150.17, 143.66, 131.08, 124.68, 119.60, 115.79, 45.82, 35.57, 27.51, 16.03, 13.93.; IR (KBr, cm^{-1}): 3377, 2977, 2948, 1736, 1615, 1467, 1319, 1246, 1159, 1120, 1022.

*(d) 6-(Allyloxy)-3,3,5,8-tetramethylhydocoumarin (**6**)*

6-Hydroxy-3,3,5,8-tetramethylhydrocoumarin **5** (13.8 g, 62.6 mmol), potassium carbonate (18.1 g, 131 mmol) (Ajax Finechem, analytical grade) and sodium iodide (0.20 g, 1.38 mmol) (Ajax Finechem, analytical grade) were suspended in acetone (70 mL). Allyl bromide (15.9 g, 131 mmol) (Sigma-Aldrich, 98-99%) was added to the reaction mixture in one portion while stirring at room temperature. The suspension was then heated to 60 °C while stirring for 5.5 days under argon. Additional allyl bromide (1.66 g) was added after 1, 2 and 3 days. After cooling of the reaction mixture to room temperature and evaporation of the solvent *in vacuo,* the crude title compound **6** was recovered as a dark yellow oil. The obtained residue

was then dissolved in dichloromethane and washed with water (70 mL). The aqueous phase was further extracted with dichloromethane (2 × 35 mL) and the pooled organic layers washed with water (2 × 35 mL) before drying over $MgSO_4$. Filtration through celite and evaporation of the solvent *in vacuo* afforded the crude coumarin **6** as an orange/brown residue. Column chromatography (dichloromethane) gave the pure product **6** (13.5 g, 83%) as a pale yellow solid. ^{1}H NMR (300 MHz, $CDCl_3$) δ: 6.63 (s, 1H), 6.13–6.00 (m, 1H), 5.41 (dd, 1H, *J* = 1.5 Hz, *J* = 17.3 Hz), 5.28 (dd, 1H, *J* = 1.5 Hz, *J* = 10.6 Hz), 4.49 (d, 2H, *J* = 5.3 Hz), 2.56 (s, 2H), 2.35 (s, 3H), 2.26 (s, 3H), 1.46 (s, 6H); ^{13}C NMR (75.5 MHz, CDCl3) δ: 168.93, 153.58, 144.16, 133.88, 131.47, 124.54, 123.11, 117.44, 113.73, 70.15, 46.35, 36.07, 27.96, 16.88, 14.46; IR (KBr, cm^{-1}): 2956, 1760, 1607, 1467, 1323, 1276, 1246, 1192, 1163, 1124, 1029, 916.

(e) 7-Allyl-6-hydroxy-3,3,5,8-tetramethylhydrocoumarin (7)

6-(Allyloxy)-3,3,5,8 tetramethylhydocoumarin **6** (13.2 g, 50.8 mmol) was dissolved in dichloromethane and the solution stirred at 0 °C under argon. An ice cold 1.0 M solution of boron trichloride in heptane (85 ml, 85 mmol) (Sigma-Aldrich) was added dropwise *via* a dropping funnel to the reaction mixture over a 10 min period. Stirring was continued at 0 °C for an additional 4 h and then at room temperature for 12 h. Ice cold saturated sodium bicarbonate solution (*ca*. 85 mL) was then added to the reaction mixture with vigorous stirring over an ice/water bath. The organic phase was separated and washed with water (2 × 45 mL), dried over $MgSO_4$, filtered and evaporated under vacuum to afford the crude **7** as a yellow oil. Column chromatography (dichloromethane) gave the pure product **7** (10.4 g, 79%) as a yellow solid. ^{1}H NMR (300MHz, $CDCl_3$) δ: 6.00–5.87 (m, 1H), 5.16-5.064 (m, 2H), 4.80 (s, 1H), 3.43 (d, 2H, *J* = 5.7 Hz), 2.55 (s, 2H), 2.35 (s, 3H), 2.22 (s, 3H), 1.46 (s, 6H); ^{13}C NMR (75.5 MHz, $CDCl_3$) δ: 168.84, 149.41, 143.85, 135.05, 129.32, 123.43,

123.38, 120.11, 116.51, 46.14, 35.69, 31.43, 27.79, 14.48, 12.36; IR (KBr, cm^{-1}): 3405, 3010, 2958, 1743, 1636, 1444, 1287, 1261, 1214, 1190, 1127, 1038, 902.

*(f) 7-Allyl-6-(methoxymethoxy)-3,3,5,8-tetramethylhydrocoumarin (**8**)*

7-Allyl-6-hydroxy 3,3,5,8-tetramethylhydrocoumarin **7** (10.1 g, 38.8 mmol) was added to a solution of ethyldiisopropylamine (43 mL, 243 mmol) (Alfa-Aesar, 99%) in dry dichloromethane (*ca*. 145 mL) while stirring under an argon. The reaction flask was then cooled in an ice/water bath before adding dropwise, ice cold chloromethyl methyl ether (14.7 mL, 170 mmol) (Sigma-Aldrich, technical grade) over a 30 min period. One hour after the addition was complete, the reaction mixture was warmed to room temperature and stirring was continued for an additional 5 h. The crude mixture was then diluted with dichloromethane (100 mL), washed with ice cold 3 M hydrochloric acid (2 × 20 mL), ice cold water (2 × 50 mL), dried over $MgSO_4$ and filtered. The solvent was removed under reduced pressure and the residue purified by column chromatography (ethyl acetate/light petroleum, 1:4) to give the methoxymethyl protected product **8** as a white solid (10.9 g, 92%). ^1H NMR (300 MHz, $CDCl_3$) δ: 5.97–5.84 (m, 1H), 5.04 (dd, 1H, *J* = 1.9 Hz, *J* = 10.2 Hz), 4.93 (dd, 1H, *J* = 1.9 Hz, *J* = 18.5 Hz), 4.87 (s, 2H), 3.60 (s, 3H), 3.48 (d, 2H, *J* = 5.6 Hz), 2.54 (s, 2H), 2.40 (s, 3H), 2.21 (s, 3H), 1.44 (s, 6H); ^{13}C NMR (75.5 MHz, CDCl3) δ: 173.07, 168.53, 151.54, 146.44, 136.06, 131.41, 129.32, 126.93, 124.61, 115.59, 100.17, 57.71, 46.04, 35.82, 31.72, 27.66, 15.89, 12.47; IR (KBr, cm^{-1}): 3013, 2971, 1764, 1636, 1435, 1388, 1248, 1154, 1168, 1154, 1041, 982.

*(g) 7-(3-Hydroxypropyl)-6-(methoxymethoxy)-3,3,5,8-tetramethylcoumarin (**9**)*[2]

7-Allyl-6- (methoxymethoxy)-3,3,5,8-tetramethylhydrocoumarin **8** (10.5 g, 34.5 mmol) was dissolved in anhydrous tetrahydrofuran (*ca*. 280 mL) and the solution stirred vigorously for 20 min while bubbling argon through it. The reaction mixture was then cooled in an ice/water

bath before adding a large excess of 1.0 M borane tetrahydrofuran complex solution (1 M) in tetrahydrofuran (56 mL, 56 mmol) (Sigma-Aldrich) in a dropwise manner over a 30 min period under argon. After the addition was complete, the mixture was stirred at 0 °C for 20 min under an inert atmosphere. The reaction mixture was then warmed to room temperature and stirring was continued for an additional 12 h under argon. Upon cooling of the reaction mixture to 0 °C in an ice bath, 3 M sodium hydroxide solution (10 mL, 30 mmol) and 30% hydrogen peroxide solution (10 mL, 86.8 mmol) were simultaneously added dropwise to the reaction mixture from separate dropping funnels while stirring was continued. The reaction was continued for 5 min before removing the ice bath. Stirring was continued under ambient atmosphere at room temperature for an additional 1 h. The crude mixture was diluted with ethyl acetate (140 mL) and then transferred into a separation funnel where water (*ca*. 30 mL) was added. Hydrochloric acid (3 M) was added to reduce the solution *ca*. pH 5. When bubbling was significantly reduced, but still present, the water layer was removed, and the organic layer was washed with water (2 × 35 mL), brine (35 mL), dried over $MgSO_4$ and filtered. The solvent was then evaporated *in vacuo* and the residue purified by column chromatography (toluene) to give the coumarin **9** as a white solid (4.88 g, 44%). ^{1}H NMR (300 MHz, $CDCl_3$) δ: 4.81 (s, 2H), 3.63 (s, 3H), 3.57 (t, 2H, *J* = 6.0 Hz), 2.81 (t, 2H, *J* = 7.1 Hz), 2.55 (s, 2H), 2.38 (s, 3H), 2.24 (s, 3H), 1.76 (m, 2H), 1.44 (s, 6H); ^{13}C NMR (75.5 MHz, $CDCl_3$) δ: 168.41, 151.45, 146.68, 133.37, 129.02, 126.54, 123.93, 100.19, 61.85, 57.91, 45.95, 35.74, 32.22, 27.61, 23.42, 15.85, 12.39; IR (KBr, cm^{-1}): 3417, 2952, 1766, 1666, 1600, 1470, 1417, 1318, 1246, 1159, 1040, 980.

*(h) 3-(6-(Methoxymethoxy)-3,3,5,8-tetramethylcoumarin-7-yl) propyl 4-methylbenzenesulfonate (**10**).*

7-(3-Hydroxypropyl)-6-(methoxymethoxy)-3,3,5,8-tetramethylcoumarin **9** (4.50 g, 14.0 mmol) was dissolved in dry pyridine (9 mL) and dry dichloromethane (30 mL) and the stirred

solution cooled in an ice/water bath under an argon atmosphere. *p*-Toluenesulfonyl chloride (2.93 g, 15.3 mmol) (Sigma-Aldrich, ≥ 98%) was added in one portion and the reaction continued under inert gas for 3 h at 0 °C and then for an additional 12 h at room temperature. The crude mixture was transferred to a separation funnel and diluted with ice (*ca*. 70 g) before adding dichloromethane (140 mL) and ice-cold 3 M hydrochloric acid (70 mL). The organic layer was separated and then further washed with water (2 × 70 mL), ice-cold 3M hydrochloric acid solution (2 × 70 mL), dried over $MgSO_4$ and filtered. Drying *in vacuo* left a pale-yellow oil that was purified by column chromatography (ethyl acetate/light petroleum, 1:2) to give the title compound **10** as an off-white solid (2.66 g, 40%). ^{1}H NMR (300 MHz, $CDCl_3$) δ: 7.80 (d, 2H, *J* = 8.1 Hz), 7.35 (d, 2H, *J* = 8.1 Hz), 4.85 (s, 2H), 4.10 (t, 2H, *J* = 6.0 Hz), 3.57 (s, 3H), 2.71 (t, 2H, *J* = 7.9 Hz), 2.54 (s, 2H), 2.45 (s, 3H), 2.34 (s, 3H), 2.07 (s, 3H), 1.84 (m, 2H), 1.43 (s, 6H); ^{13}C NMR (75.5 MHz, $CDCl_3$) δ: 172.52, 168.01, 151.45, 144.48, 132.87, 132.28, 129.54, 128.89, 127.61, 126.29, 123.23, 99.75, 70.10, 57.29, 45.49, 35.32, 28.55, 27.14, 27.61, 23.43, 21.36, 15.48, 11.97.

*(i) 7-(3-Azidopropyl)-6-(methoxymethoxy)-3,3,5,8-tetramethylcoumarin (**11**)*

To a solution of 3-(6-(methoxymethoxy)-3,3,5,8-tetramethylcoumarin-7-yl) propyl 4-methylbenzenesulfonate **10** (2.30 g, 4.83 mmol) in *N,N*-dimethylformamide (20 mL) and water (13 mL), sodium azide (2.70 g, 42 mmol) (Alfa-Aesar, 99%) was added in one portion with stirring at room temperature. The obtained suspension was warmed to 60 °C in an oil bath and stirred under an argon atmosphere for 16 h. The mixture was concentrated *in vacuo* (bath temperature not exceeding 60 °C) to leave an off-white slurry which was then suspended in ice-cold ethyl acetate (75 mL) to precipitate excess sodium azide, which was then removed by filtration. The filtrate was evaporated *in vacuo* to give the substituted azide **11** as a white solid (1.27 g, 76%) and was used for subsequent steps without the need for purification. ^{1}H NMR (300 MHz, $CDCl_3$) δ: 4.90 (s, 2H), 3.62 (s, 3H), 3.36 (t, 2H, *J* = 6.4

Hz), 2.76 (t, 2H, *J* = 7.9 Hz), 2.55 (s, 2H), 2.38 (s, 3H), 2.24 (s, 3H), 1.81-1.73 (m, 2H), 1.45 (s, 6H); ^{13}C NMR (75.5 MHz, CDCl3) δ: 168.52, 151.82, 146.47, 133.10, 129.21, 126.71, 123.68, 100.17, 57.72, 51.55, 45.93, 35.75, 28.98, 27.58, 24.87, 15.95, 12.46.

*(l) 7-(3-Azidopropyl)-6-hydroxy-3,3,5,8-tetramethylhydrocoumarin (**12**)*

To a stirred solution of 7-(3-azidopropyl)-6-(methoxymethoxy)-3,3,5,8-tetramethylhydrocoumarin **11** (0.900 g, 2.59 mmol) in anhydrous 2-propanol (225 mL), carbon tetrabromide (180 mg, 0.537 mmol) (Sigma-Aldrich, 99%) was added in one portion while stirring under argon. The reaction mixture was heated to *ca*. 80 °C and stirring was continued for 12 h. The reaction mixture was evaporated *in vacuo* and the crude yellow oil was purified *via* column chromatography (ethyl acetate/light petroleum, gradient of 0-30% ethyl acetate) to afford the title compound **12** as a white solid (550 mg, 70%). ^{1}H NMR (300 MHz, $CDCl_3$) δ: 5.19 (s, 1H), 3.36 (t, 2H, *J* = 6.4 Hz), 2.75 (t, 2H, *J* = 7.1 Hz), 2.55 (s, 2H), 2.35 (s, 3H), 2.23 (s, 3H), 1.87-1.79 (m, 2H), 1.46 (s, 6H); ^{13}C NMR (75.5 MHz, $CDCl_3$) δ: 168.72, 149.15, 143.87, 129.05, 125.15, 123.34, 119.62, 50.97, 46.17, 35.68, 28.08, 27.84, 23.55, 14.69, (KBr, cm^{-1}): 3417, 2964, 2111, 1731, 1412, 1303, 1260, 1188, 1031, 801.

Synthesis of linker that is not electrochemically-cleavable

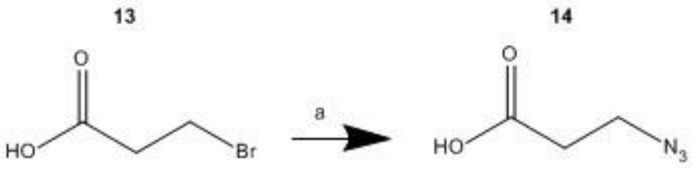

Supplementary Figure 4. Synthesis of the linker molecule 14. a. NaN_3, 33%

3-Azidopropionic acid **14** was synthesised from a previously reported method described by Srinivasan *et al*.[4]. Briefly, a solution of 3-bromopropionic acid (**13**) (7.824 g, 50.1 mM) (Sigma-Aldrich, 97%) and sodium azide (6.641 g, 100 nM) in redistilled acetonitrile (80.2 mL) was refluxed for 4 h at 100 °C under an argon atmosphere. Acetonitrile was then removed *in vacuo* and the resulting residue was redissolved in redistilled ethyl acetate and

washed with 0.1 M hydrochloric acid (2 × 10 mL), MilliQ water (2 x 10 mL) and brine (2 × 10 mL). The organic solution was dried using anhydrous sodium sulfate and filtered. All remaining solvent was removed *in vacuo* to afford compound **14** as a dark, orange-brown oil (2.19 g, 33 %). 1H NMR (300 MHz, CDCl3) δ: 3.591 (t, 2H, *J* = 2.00 Hz), 2.636 (t, 2H, *J* = 2.10 Hz); 13C NMR (75.5 MHz, CDCl3) δ: 176.81, 46.48, 33.78; IR (NaCl, cm^{-1}): 3187.40, 2104.89, 1716, 69, 1403.86, 1243.07, 1175.65.

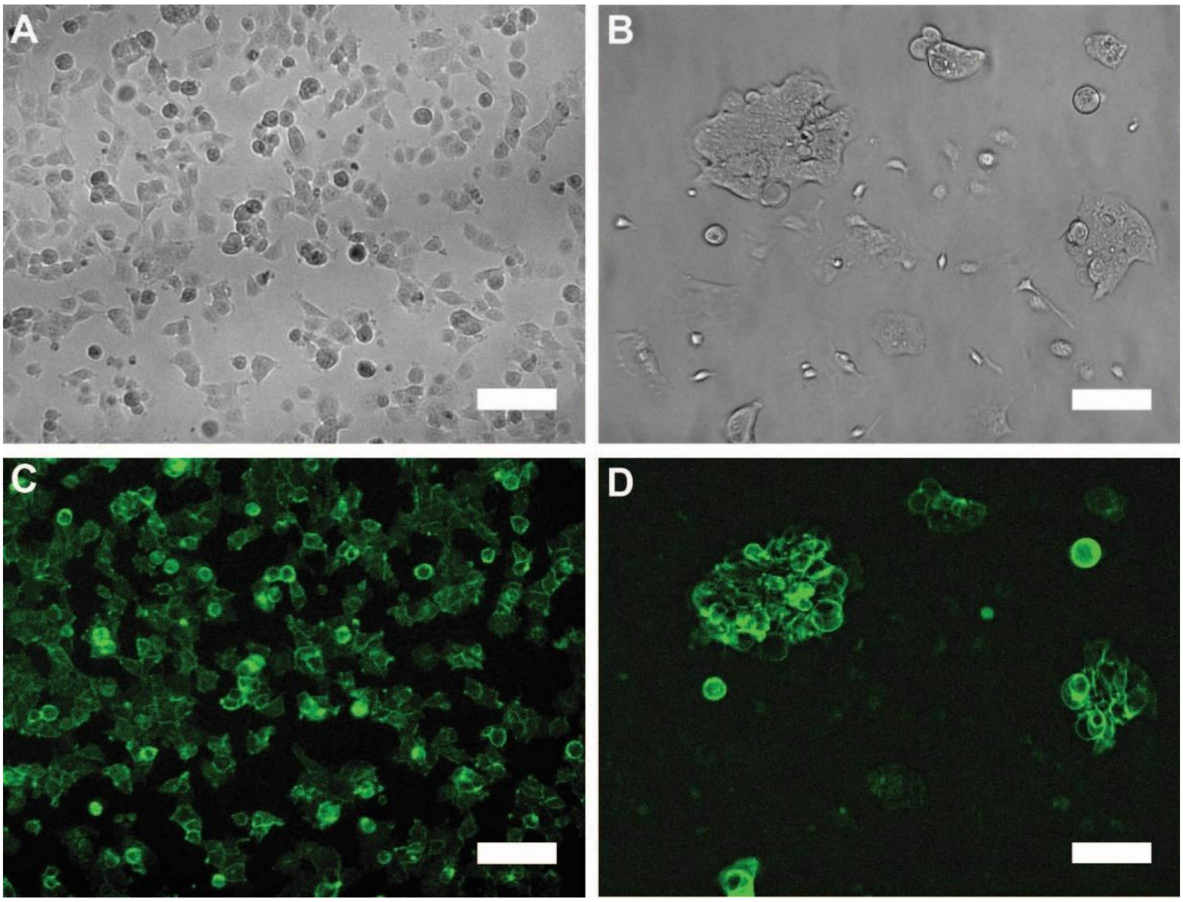

Supplementary Figure 5. EpCAM-expression of target cells. Brightfield images of (A) MCF-7 cells and (B) lung tumor cells. Fluorescence microscopy images of (C) MCF-7 cells and (D) lung tumor cells stained with a FITC-conjugated anti-EpCAM to confirm EpCAM upregulation within the target cells. Scale bar = 100 µm.

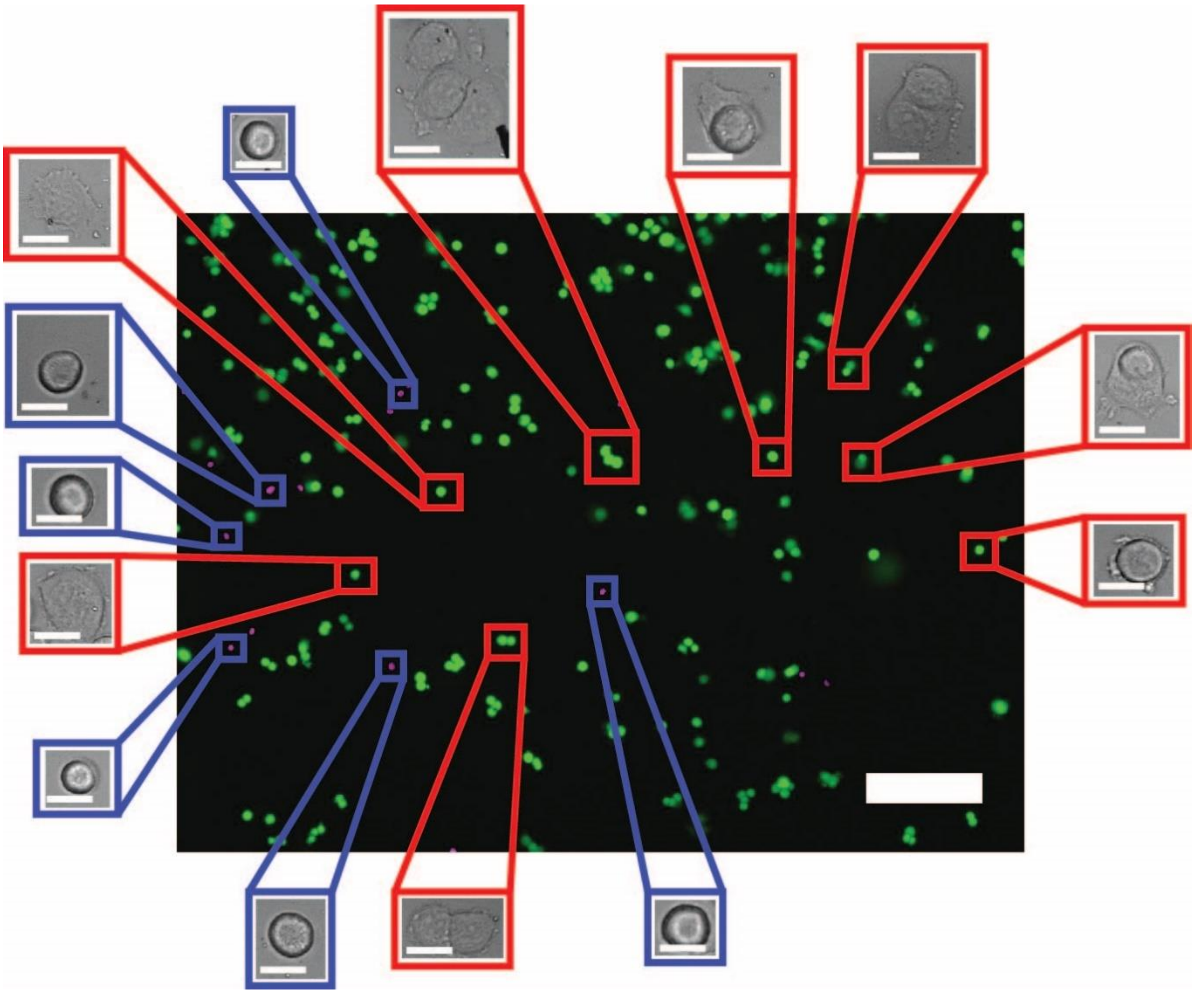

Supplementary Figure 6. The morphologies of target cells *versus* contaminating cells on the surface. Microscopy images of anti-EpCAM-modified surface incubated with a cocktail containing MCF-7 cells (25% of 6.57 x10^5 total cells in suspension) and HeLa cells (75% of 6.57 × 10^5 total cells in suspension). MCF-7 cells (green cells in the fluorescence micrograph, 68 ± 20% of total cells on the surface) and HeLa cells (magenta cells in the fluorescence micrograph, 8 ± 7% of total cells on the surface) with high magnification brightfield images showing that MCF-7 cells are spread on the anti-EpCAM-modified silicon surface whilst HeLa cells maintain a spherical shape on the anti-EpCAM-modified silicon surface. Scale bar in fluorescence micrograph = 100 µm. Scale bar in brightfield images of single cells = 10 µm.

Optimising electrochemical release

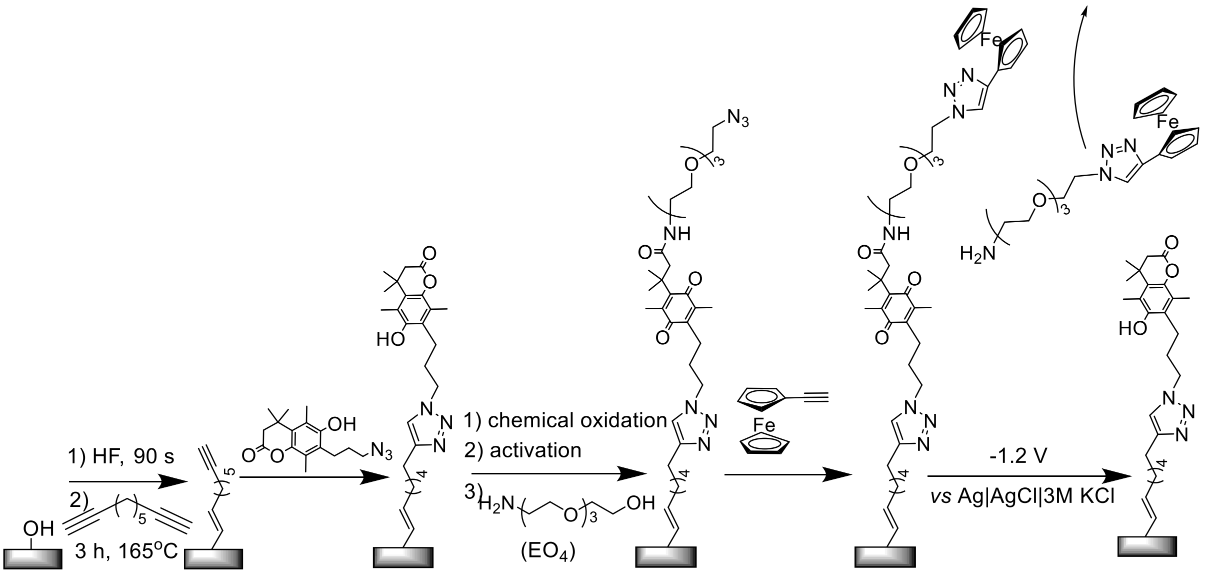

Supplementary Figure 7. Fabricating surface to confirm polarisation window. A schematic showing how a redox receptor molecule (ferrocene) was tethered to a silicon surface *via* a switchable component. A negative bias *vs* Ag|AgCl can be applied to the surface to initiate switching of the switchable component to remove the redox reporter molecule from the surface.

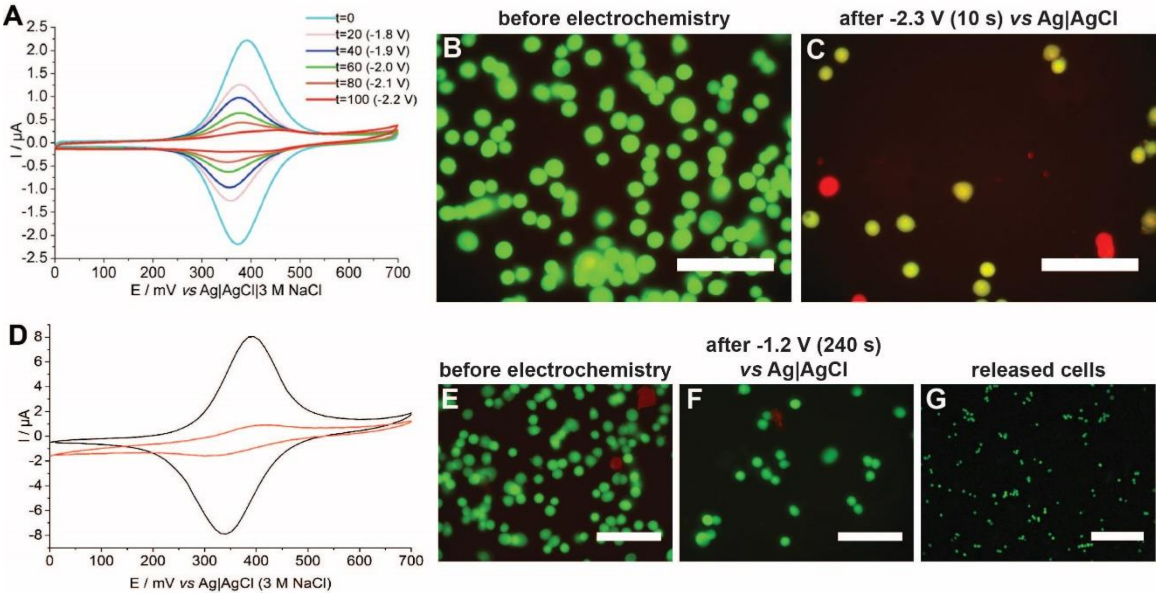

Supplementary Figure 8. Optimising the polarisation window for cell release. (A) Representative cyclic voltammograms of a ferrocene-terminated cleavable surface *via* an amine-OEO4-azide molecule after 20 s intervals of negative potentials from -1.8 V to -2.2 V *vs* Ag|AgCl (3 M NaCl) in -0.1 V increments. After 100 s of total polarisation time, the measured ferrocene coverage, Γ, decreased to 1.38×10^{-11} mol.cm^{-2} from 1.89 x 10^{-10} mol cm^{-2}. Peak fwhm decreased initially being 110, 109 and 107 mV for 0, 20 and 40 s, respectively but then increased to 118, 133 and 159 mV for 60, 80 and 100 s, respectively. CVs were acquired in 1.0 M $HClO_4$ (ν

= 50 mV.s^{-1}). Fluorescence micrographs of MCF-7 cells stained with a LIVE/DEAD stain on the final surface (B) before electrochemistry and (C) after -2.3 V for 10 s *vs* Ag|AgCl. Scale bar is the same for both images = 100 µm. (D) Cyclic voltammograms of the same surface in A before electrochemistry (black line) and after -1.2 V for 240 s *vs* Ag|AgCl (3 M NaCl) (red line). After 240 s of total polarisation time, the measured ferrocene coverage, Γ, decreased to 5.70 × 10^{-12} mol cm^{-2} from 4.42 × 10^{-11} mol cm^{-2}. Peak fwhm increased from 116 mV to 140 eV and ΔEp increasing from 53 mV to 107 eV. CVs were acquired in 1.0 M $HClO_4$ (ν = 100 mV s^{-1}). Fluorescence micrographs of MCF-7 cells stained with a LIVE/DEAD stain on the final surface within Supplementary Figure 3 (E) before electrochemistry and (F) after -1.2 V for 240 s *vs* Ag|AgCl. Scale bar is the same for both images = 100 µm. (G**)** Fluorescence micrographs of MCF-7 cells released from the surface and stained with a LIVE/DEAD stain. Scale bar = 250 µm.

Modifying the azide-terminated OEO surface, used to characterise the conjugation of the antifouling molecule on the surface, with an alkyne-terminated redox species, ferrocene (Supplementary Figure 7), enabled us to determine the negative polarisation window required to initiate electrochemical cleavage of the monolayer. It was discovered that -2.2 V *vs* Ag|AgCl for 20 s allowed electrons to transfer to the electrochemically-cleavable quinone. The two electron-two proton reduction resulted in the lactonisation of the benzoquinone back to the hydroquinone, cleaving the layer tethered above it. This resulted in a 93% decrease of the ferrocene signal (Supplementary Figure 8A). This polarisation window was applied to an anti-EpCAM-modified cleavable surface after it had been preloaded into a customised electrochemical chamber before capturing and staining MCF-7 cells on it. Capturing the MCF-7 cells on the surface after the surface had been loaded into the customised electrochemical chamber ensured that the cells were not dried out and improved their viability prior to their electrochemical release from the surface (Supplementary Figure 8B). However, Supplementary Figure 8C reveals that this polarisation window was detrimental to the membrane of the cells, where released cells were significantly harmed throughout the process. Supplementary Figure 8D reveals that applying -1.2 V for 240 s *vs* Ag|AgCl resulted in an 87% decrease in the ferrocene signal. Supplementary Figure 8G reveals that the cell

membranes of the released cells remained intact after applying -1.2 V for 240 s *vs* Ag|AgCl, highlighting the suitability of this polarisation regime to release cells.

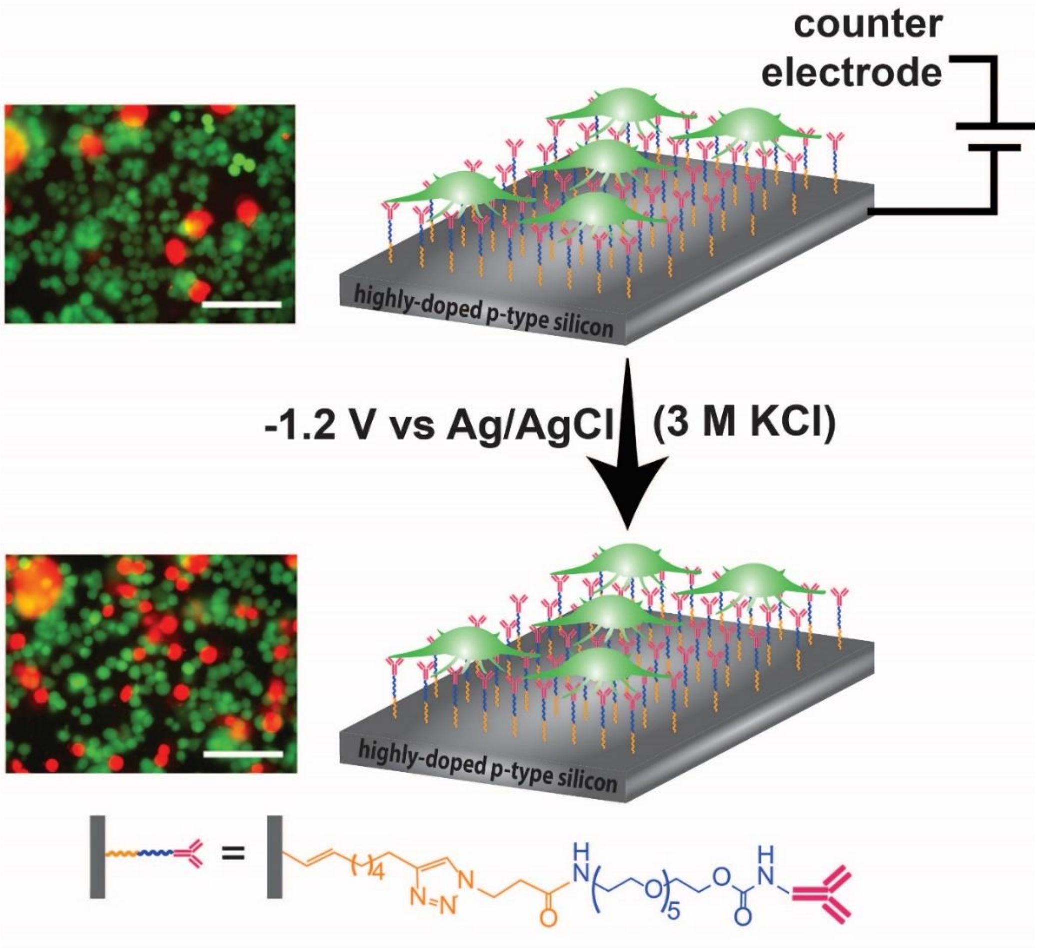

Supplementary Figure 9. Effect of potential on non-cleavable surface. A schematic illustrating the fabrication of a surface that is not electrochemically switchable and LIVE/DEAD stains of the cells on the anti-EpCAM-modified silicon surface both before and after a negative bias was applied to the surface. Scale bar = 100 µm.

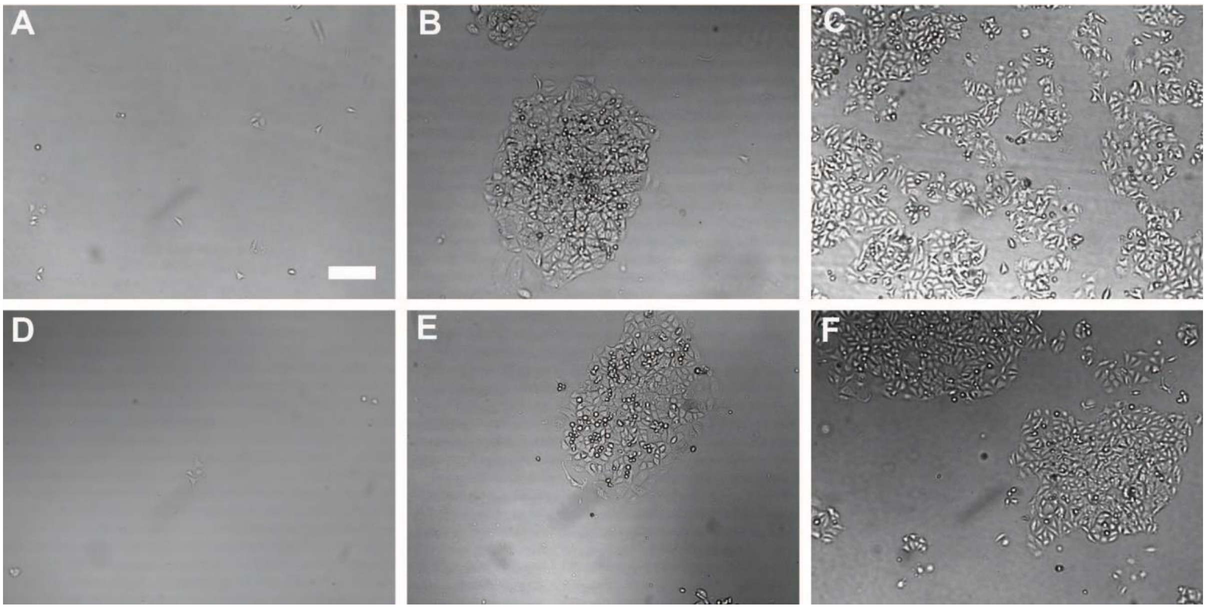

Supplementary Figure 10. Growth comparison of captured & released MCF-7 cells with cultured MCF-7 cells. Brightfield images of MCF-7 cells in culture. Captured-and-released MCF-7 cells after one day (A) and 11 days (B) from plating them on T25 flasks. Dense clusters were observed after 11 days so the cells were trypsinised and allowed to respread on the T25 flasks and imaged again three days after reseeding (C). MCF-7 cells in continuous culture were plated in T25 flasks at the same concentration as the released MCF-7 cells and imaged after one day (D) and 11 days (E) before being trypsinised, reseeded on T25 flasks and imaged after three days (F). Scale is the same for all images. Scale bar = 200 µm.

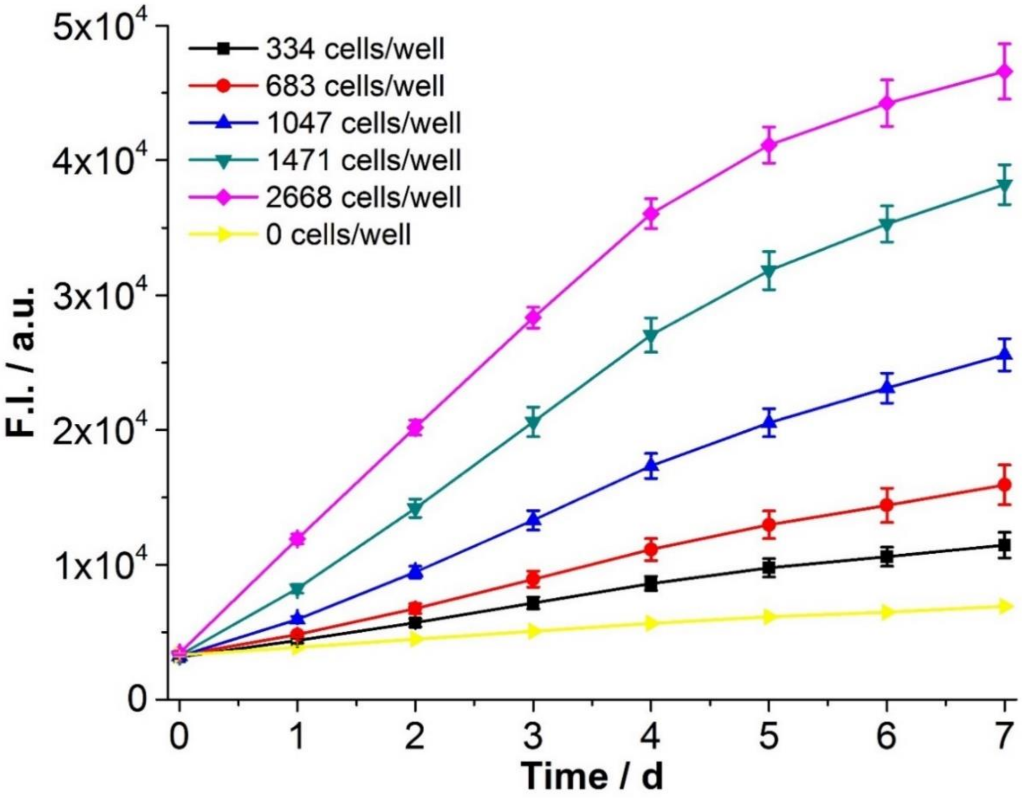

Supplementary Figure 11. Growth profile of MCF-7 cells at different densities, treated with alamarBlue®. Different concentrations of MCF-7 cells (equivalent volumes for each concentration, 200 µL) were plated, n = 7, in separate wells of a 96-well plate. AlamarBlue® (20 % v/v) was added to each well before fluorescence measurements were recorded daily for 7 days. An excitation wavelength of 544 nm, emission wavelength range of 590-610 nm and a gain factor = 750 was used. Error bars indicate 95% confidence interval.

Supplementary References

1 Liotta, D., Arbiser, J., Short, J. W. & Saindane, M. A simple, inexpensive procedure for the large-scale production of alkyl quinones. *J. Org. Chem.* **48**, 2932-2933 (1983).

2 Zheng, A., Shan, D. & Wang, B. A redox-sensitive resin linker for the solid phase synthesis of C-terminal modified peptides. *J. Org. Chem.* **64**, 156-161 (1999).

3 Rohde, R. D., Agnew, H. D., Yeo, W. S., Bailey, R. C. & Heath, J. R. A non-oxidative approach toward chemically and electrochemically functionalizing Si(111). *J. Am. Chem. Soc.* **128**, 9518-9525 (2006).

4 Srinivasan, R. *et al.* High-throughput synthesis of azide libraries suitable for direct "click" chemistry and in situ screening. *Org. Biomol. Chem.* **7**, 1821-1828 (2009).

5 Linford, M. R. & Chidsey, C. E. D. Alkyl monolayers covalently bonded to silicon surfaces. *J. Am. Chem. Soc.* **115**, 12631-12632 (1993).

6 Linford, M. R., Fenter, P., Eisenberger, P. M. & Chidsey, C. E. D. Alkyl monolayers on silicon prepared from 1-alkenes and hydrogen-terminated silicon. *J. Am. Chem. Soc.* **117**, 3145-3155 (1995).

7 Ciampi, S. *et al.* Functionalization of acetylene-terminated monolayers on Si(100) surfaces: A click chemistry approach. *Langmuir* **23**, 9320-9329 (2007).

8 Ciampi, S. *et al.* Silicon (100) electrodes resistant to oxidation in aqueous solutions: An unexpected benefit of surface acetylene moieties. *Langmuir* **25**, 2530-2539 (2009).

9 Ng, C. C. A., Ciampi, S., James, M., Harper, J. B. & Gooding, J. J. Comparing the reactivity of alkynes and alkenes on silicon (100) surfaces. *Langmuir* **25**, 13934-13941 (2009).

10 Ciampi, S., Le Saux, G., Harper, J. B. & Gooding, J. J. Optimization of click chemistry of ferrocene derivatives on acetylene-functionalized silicon(100) surfaces. *Electroanalysis* **20**, 1513-1519 (2008).

11 Ng, C. C. A., Ciampi, S., Harper, J. B. & Gooding, J. J. Antifouling behaviour of silicon surfaces modified with self-assembled monolayers containing both ethylene glycol and charged moieties. *Surf. Sci.* **604**, 1388-1394 (2010).

12 Janolino, V. G. & Swaisgood, H. E. Analysis and optimization of methods using water-soluble carbodiimide immobilization of biochemicals to porous-glass. *Biotechnol. Bioeng.* **24**, 1069-1080 (1982).

13 Ciampi, S., Bocking, T., Kilian, K. A., Harper, J. B. & Gooding, J. J. Click chemistry in mesoporous materials: Functionalization of porous silicon rugate filters. *Langmuir* **24**, 5888-5892 (2008).

14 Collman, J. P., Devaraj, N. K., Eberspacher, T. P. A. & Chidsey, C. E. D. Mixed azide-terminated monolayers: A platform for modifying electrode surfaces. *Langmuir* **22**, 2457-2464 (2006).
